# Case Management for People with Acquired Brain Injury with Complex Problems (Part 2): Process Evaluation of a One-Group Trial

**DOI:** 10.5334/ijic.8650

**Published:** 2025-07-07

**Authors:** Annemarie P. M. Stiekema, Desiree Bierlaagh, Mireille Donkervoort, Natska Jansen, Kitty H. M. Jurrius, Judith Zadoks, Caroline M. Van Heugten

**Affiliations:** 1 Department of Psychiatry and Neuropsychology, School of Mental Health and Neuroscience, Maastricht University, Maastricht, The Netherlands; 2 Limburg Brain Injury Center, Maastricht University, Maastricht, The Netherlands; 3 Self-employed, Houten, The Netherlands; 4 Health Care and Social Work Division, Windesheim University of Applied Sciences, Almere, The Netherlands; 5 Mevrouw Slimmer Werken Social Innovation in Health Care and Well-Being, Drogteropslagen, Netherlands; 6 Brain Injury Team, Overijssel, The Netherlands; 7 In-Tussen Foundation, Utrecht, the Netherlands; 8 BreinDok Innovation in Care, Utrecht, The Netherlands; 9 Department of Neuropsychology and Psychopharmacology, Faculty of Psychology and Neuroscience, Maastricht University, Maastricht, The Netherlands

**Keywords:** acquired brain injury, caregivers, integrated care, psychosocial, long-term, feasibility

## Abstract

**Introduction:**

People with acquired brain injury (PwABI) and their families may face psychosocial problems and unmet needs. We assessed the feasibility of Case Management (CM) for PWABI in the Netherlands which aims to facilitate access to and integration of health care and social services for people with complex problems.

**Methods:**

We evaluated if CM was delivered according to plan, if participants and case managers (CMrs) were satisfied with CM, and which factors affected implementation. Data were collected using evaluation forms, logs and minutes, and interviews.

**Results:**

Twenty-eight PwABI, 13 caregivers, 17 CMr and all 3 project leaders participated. CM key elements were applied. PwABI and their caregivers generally expressed satisfaction with CM, though for some it did not meet expectations. CMrs valued the continuous involvement and the ability to support family. Facilitators were CMr working independent from existing care services and the diversity in expertise in the CMr teams. Barriers were imposed when a region offers medical stroke care but no integrated long-term services after brain injury.

**Discussion:**

It is important to ensure clear communication with PwABI and their family regarding the roles of CMr, as well as managing and aligning expectations.

**Conclusion:**

CM after ABI is feasible and warrants further research.

## Introduction

Acquired brain injury (ABI) such as stroke and traumatic brain injury can lead to persistent physical, communicative, cognitive, emotional, and behavioural consequences that negatively impact daily functioning and well-being [1,2,3]. Timely access to health care and social services that understand the impact of ABI is essential for psychosocial well-being [4,5,6]. A key coordinator who proactively integrates appropriate care services to address diverse needs according to collaboratively formulated long-term strategies is essential [7,8,9,10].

We therefore developed case management (CM) for people with acquired brain injury (PWABI) in the Netherlands along the framework proposed by Lukersmith and colleagues [16] and descriptions of CM for people with dementia in the Netherlands [11]. The new intervention was delivered in two separate studies. A randomized controlled trial on ‘early’ CM (i.e., from hospital visit or admission onwards) compared to usual care (for design, see [11]). In this study, the benefits of early CM were not shown, but access to CM was hindered due to unsuccessful monitoring of PwABI after hospital discharge [11].

In the second study, an explorative uncontrolled trial, we evaluated the outcomes and feasibility of ‘late’ CM for PwABI with complex problems affecting multiple life domains and their family. This paper describes the results of the process evaluation of CM for PwABI. The outcome evaluation applying patient-reported outcome measures, is presented the parallel paper (part 1). This process evaluation assessed the feasibility of CM. These results can be used to interpret the results of the outcome analyses, and for treatment optimization and implementation.

## Methods

### Study design

This process evaluation was conducted alongside an uncontrolled trial examining CM for PwABI and their family with unmet care needs in multiple life domains. The trial took place between November 2019 and December 2022 in three regions in the Netherlands. Participants were followed up every six months for 18 to 24 months until the end of data collection in December 2022. This evaluation follows the framework of Saunders and colleagues [12] providing the elements of a process evaluation plan as described in Table 1.The study protocol was approved by the Ethics Review Committee Psychology and Neuroscience at Maastricht University (ERCPN-212_01_09_2019). All participants gave informed consent. For more detailed information on the study design, see the parallel paper presenting the outcome evaluation of CM (Part 1) [13].

**Table 1:** Process evaluation outcome measures.


ELEMENT	OPERATIONALISATION	PROCESS EVALUATION QUESTIONS	EVALUATION FORMS PARTICIPANTS	INTER-VIEWS PARTICIPANTS	INTER-VIEWS AND FOCUS GROUPS CASE MANAGERS	COMMUNICATION REPORTS

**Fidelity**	The extent to which the intervention was implemented consistent with the underlying theory and philosophy of CM	- Did CM follow the stepped care principle? - Did the CM have a continuous professional relationship with the client and family member?	x	x	x	x

**Dose delivered**	The extent to which the CM elements were provided to participants	- What intervention components were delivered?	x	x	x	x

**Dose received – exposure**	The extent to which participants were engaged in the CM activities	- To what extent did CM support participants self-management (i.e., only taking over activities when the participant is too overwhelmed)?		x	x	x

**Dose received – satisfaction**	Participants and CMs’ satisfaction with CM (expected to be >7 on a scale 1–10)	- What is the overall opinion and the opinion of the value of participants of CM? - What is the overall opinion of the CM about the intervention and its value?	x	x	x	

**Reach**	The proportion of the target audience that participated in the intervention	- How many participants were supported by a CM? - What were participants’ reasons for not seeking contact with a CM?	x			

**Recruitment**	The procedures used for recruitment and engagement of participants and CM.	- What procedures were followed for recruitment and engagement? - What where the barriers and facilitators for recruiting participants and CM? - What where the barriers and facilitators for keeping participants and CM engaged?			x	

**Context**	Factors in the organisation, community, social/political context, or other situational issues that potentially affect implementation or outcomes.	- What barriers and facilitators influenced delivery of CM?			x	x


### Participants

Adults with ABI and their loved one were included if the PwABI received CM due to experienced unaddressed problems due to a neurologically confirmed ABI, had sufficient command of the Dutch language and were willing and able to provide informed consent. During recruitment, it appeared that CMrs also provided care to people with comorbid neurodegenerative disease or without objectified brain injury. We decided to include those in the study as well. No exclusion criteria were held.

### Case management intervention and pilot

The development of CM was part of a national pilot of the Dutch Ministry of Health, Welfare and Sport to facilitate access to and integration of health care and social services to help PwABI and family members regain control over their lives. The CM intervention and the pilot are described in detail in part 1 of our study [13].

### Procedure

PwABI who received CM were eligible for participation in the study if they met all criteria. If the PwABI was interested in participating in the study, the CMr forwarded their contact details to the research team. They were contacted by the researcher who explained the procedures in full, collected written informed consent and planned the baseline assessment. After the baseline assessment, participants were provided with CM and they were able to utilize it for the entire duration of the study. Assessments took place every 6 months and were administered via digital questionnaires if possible, and on paper or via telephone based on the abilities and preference of the participants. Depending on the time of inclusion, participants were followed up for 18 or 24 months until the end of data collection in December 2022 (predefined study end date by the subsiding party).

### Data collection

Several sources were used to report on the process evaluation outcomes. See Table 1 for an overview.

Participants completed an evaluation form to provide their experiences with CM after 6 months and at study end covering the following themes: type of support received, experiences, helpful elements, elements missed, and satisfaction with the support (evaluation form available from the author upon request). Nine PwABI and nine family members were purposively selected (three from each region) to participate in interviews addressing experienced problems, received support, addressed needs, involvement of CMr with PwABI and/or relatives, helpful elements and satisfaction (topic guide available from the author upon request), to include a diverse range of demographic and injury-related characteristics.

#### Case managers

Nine CMrs were interviewed in May/June 2020 on the following topics: CMr tasks and skills, preparation and education, team work and pitfalls, barriers, and facilitating factors in collaborating with other professionals, experienced outcomes and added value, and job satisfaction. Twelve CMrs (incl. 4 of the previously interviewed CMrs) participated in one of two focus groups in March 2022 discussing CMr roles, delivery, experiences, collaboration with others and satisfaction. The project team members were interviewed May 2021 covering tasks and activities, experiences, collaboration, management and procedures, implementation, roles and tasks of the CMr, and suggestions for changes (topic guides available from the author upon request).

### Data analysis

Quantitative data were descriptively analysed using IBM SPSS version 27. Responses to open-ended questions of the evaluation forms, and minutes of meetings were thoroughly read and summarized under corresponding process evaluation themes by the first author (APMS). The first author also coded and analysed interview transcripts using a pragmatic deductive approach [14], categorizing the codes into corresponding process evaluation themes. A constant comparison analysis was applied to the transcripts of focus groups [15], where codes were applied to smaller units of text (open coding), then categorized (axial coding) and finally summarized in broader themes (selective coding). Coding and categorization were done by two researchers (APMS and Norma Jansen-Veugen) who reached a consensus after discussions on any differences in interpretation. The categories and themes were summarized with the corresponding process evaluation theme.

## Results

### Response

Out of 62 PwABI and 36 family members included, 28 (45%) PwABI and 13 (36%) family members completed the final assessment (see Table 2 for demographic information on the complete sample). To collect interview data from three PwABI and three family members per region, 16 PwABI and 12 family members were invited to participate until a total of nine PwABI and nine caregivers agreed.

**Table 2:** Demographic and injury-related information.


	PWABI (n = 62)	FAMILY MEMBERS (n = 36)

Age, mean years ± SD [range]	53.79 ± 15.08	56.67 ± 10.65

Gender, F/M	29/33	29/7

Education level, %		

Low (1–4)	19.4	13.9

Middle (5)	50.0	55.6

High (6–7)	30.6	30.6

Type of brain injury, %		

Cerebrovascular accident	33 (53.2)	

Cerebral infarction		

Intracerebral haemorrhage		

Intracranial haemorrhage		

Transient ischaemic attack	6 (9.7)	

Traumatic brain injury	17 (27.4)	

Concussion/contusion		

Other head trauma (trauma capitis)		

Other cerebrovascular disorder	1 (1.6)	

Meningitis	1 (1.6)	

Encephalitis	–	

Hypoxia	2 (3.2)	

Brain tumour	2 (3.2)	

Time since injury, mean years (SD)	5.69 ± 7.74	

Hospital admission, n (%)	44 (74.6)	

Prior brain injury, n (%)	22 (35.5)	

Living situation, %		

Alone	26.2	

With partner	37.7	

With partner and children	24.6	

With children	4.9	

With other people	6.6	

Unknown	1.6	

Relationship with PwABI, %		

Partner	66.7	

Parent	16.7	

Child	8.3	

Friend	8.3	

Living together w/ PwABI, %	77.8	


PwABI = people with acquired brain injury.

### Fidelity

The CMrs reported to assess the support needs and adjust accordingly. They often anticipated problems that the participants were not yet aware of or ready to address. The CMrs maintained contact and provided support as soon as the participants were ready. CM support was gradually decreased over time, see supplementary figure 1. On average, the case managers offered 2.4 hours support per participant per month. Most support was delivered during the first 6 months (mean 5.0 hours with a peak in the second month) which declined over time (2.5 hours at 12 months, 1.2 at 18 months and 0.6 at 24 months). These results confirm the stepped care principle where not a ‘one sits fits all’ approach is taken, but rather a personalized care approach adjusting to the needs continuously.

CMrs and interviewed participants reported that the CMr decreased contact once problems were dealt with, resolved, or when appropriate care services were in place. In some instances, they shifted to monitoring, while in others they agreed for the participant to reach out if any questions or issues arose. At study end, most participants no longer had regular contact with the CMr but stated that they could still reach out if needed. These findings confirm that a continuous professional relationship with varying levels of support intensity was offered. For quotes 1–3 regarding fidelity, see Table 3.

**Table 3:** Quotes illustrating the components of the process evaluation.


COMPONENT	QUOTE NUMBER	QUOTE

Fidelity	1	“*It’s nice, of course […], if at some point […] someone becomes self-reliant and no longer needs you, that you make yourself redundant. But with brain injury it’s like, something happens again and the balance they have [is disturbed] – and then you’re still needed again*” – CMr7

	2	“*And that’s bound to happen, because another phase will come and then you have to arrange all kinds of things again. And a healthcare system like that doesn’t change that quickly. So then I’m not going to try 10 times myself first, because that just doesn’t make sense*.” – Z034n (mother)

	3	*“With a case manager, of course you get a different relationship with her. So she knows me, and sometimes a few words were enough for her [to know what I meant]”* – Z020p (PwABI)

Dose delivered	4	*“Connecting [care providers] in time and action and making sure that the right things happen at the right time. No one else can do that. Because nobody else takes charge”* (CMr3)

	5	Example of engagement: “*She [the CMr] came to our house to get acquainted. And then she really, really just sat there for at least two hours. I think even longer. And she just sat and listened*” – Z049n (mother)

	6	Example of planning: “*That’s actually how it started, [the CMr asked: …] ‘imagine a year from now or two years from now, how would you want things to go with your mother? And that’s where we started working toward, what I wanted, and what my mother wanted. And those are then little steps that they were then going to take, and that they were then trying to achieve with me*.” – Z01n (daughter)

Dose received – exposure	7	*“I was able to take the steps to the UWV* (Netherlands Employees Insurance Agency) *[…], to do that myself. Yes, [my CMr] had given me enough ammunition so to speak, she supported me enough to just continue to do that myself”* – Z014p (PwABI)

	8	*“And then they were also like ‘well then you could start thinking about this. You don’t have to’, they said. ‘But try it for a while when you have time for it’. So I think they had a plan, like this or that, but not that they said that to me, like ‘you have to go there’. I came up with it myself.” –* Z01n (daughter)

Dose received – satisfaction	9	*“Then there is a case manager and you think – a burden falls off of you, you no longer have to proactively figure out everything yourself, there is someone who is going to take you by the hand in that process. And I experienced that as an enormous relief.”* (Z014n – partner)

	10	*“If you don’t have a case manager, someone who keeps an eye on you… For a long time I didn’t have to do anything for [PwABI and partner]. I really didn’t have much contact for months, and then suddenly it’s there again, and they know where to find you and you can get on with it right away.”* – CMr8

	11	*“Also appreciating [the process of family], and saying like: ‘Your husband or your partner has an [ABI], has a process, but you also have a process.’ And that is usually not seen [by others]. And often the process of the person it happened to is very different [from that of the partner]. The partner is generally in that grieving period.”* (CMr9)

	12	“*How feasible would it be, if you soon have 20 clients who want to call you for 10 minutes once every two weeks, and you also still have 10 new clients […]. At some point your schedule will be filled with just people you have to call every so many weeks*.” – CM2

Context – diverse backgrounds	13	“*Whatever question you get, there is always someone [another CM] who knows more about it or is more in that side of the field that you can approach*.” (CM2)

	14	“*[another cm] pretty much delivers treatment, with those conversations, and in a systematic way, I can’t do all that, so I don’t do that either. So, I do very different things, and that makes that […] the case manager is not uniform either*.” (CM8)


### Dose delivered

Interviewed CMs stated that they used all elements of CM in supporting complex cases. They view their primary responsibility as arranging appropriate services and facilitating communication between professionals. To fulfil this role, they believe it is essential to establish a trusting relationship with their clients. They achieve this by initially dedicating time to listening and assessing needs within the individual context, as well as discussing appropriate interventions. They also provide all CM elements to family members, acknowledging the impact of ABI on them and the lack of support they receive in the regular care. See quote 4.

Evaluation forms indicated that, as a group, participants also reported that CMrs delivered all elements (see Supplementary Table 1). CMr actions most frequently reported by participants (64.3–87.5%) were providing a listening ear and offering information and advice. See quote 5 for an example of engagement and quote 6 for an example of planning.

### Dose received

#### Exposure

CMrs reported to have constantly stimulated self-management by PwABI and their family. CMrs organized care only for some participants or reached out to other stakeholders when participants were unable to do so themselves. At times, they provided care that was intended to be delivered by regular care services (e.g. social work, support programs), such as when there were long waiting lists but the participants’ situation required immediate support. Some participants reported that with help of their CMr who advised them where to go and helped prepare those appointments, they were able to arrange most of their support themselves, confirming the self-management apporach. See quotes 7 and 8.

#### Satisfaction

At study end, PwABI rated CM on average 7.1 and family members rated it 7.5 which confirms their satisfaction (had to be at least 7) (see Supplementary Table 2). Those who gave the highest ratings reported that they appreciated having a CMr who understood them and could help them get back on track, and that they no longer felt alone and their self-esteem improved. PwABI and family members reported that they benefited most from the CMr offering a listening ear, acknowledging their problems and taking them seriously, and providing advice on accessing care services, as well as offering new insights and perspectives on their situation. Most PwABI and their family felt comfortable discussing anything with their CMr, who they felt was knowledgeable, had enough time for them and provided useful support (see Supplementary Table 3). Participants appreciated that the CMrs took the time to get to know them and their situation and had knowledge of the regular care system. Some mentioned that having someone to guide and support them was a relief and reduced burden. Approximately 90% of the participants would recommend CM to others which further confirms their satisfaction. See quote 9.

Participants found comfort in the CMrs continuous availability. The CMrs believe that the ability for participants to return with any new issues is a crucial aspect of CM. See quote 10.

Participants with the lowest satisfaction scores reported experiencing the process as a burden, for example because of difficulties reaching their CMr, no concrete plans being made, a perceived lack of understanding or dissatisfaction with the CMrs’ advise. About one third of those who filled out the evaluation form provided suggestions for improvement, most frequently expressing a desire for the CMr to proactively reach out to them, and some reported that the CMr did not respond to their questions asked via email. Some participants wanted the CMr to take over more and some were unclear about what they could ask the CMr. Other suggestions related to unresolved personal problems such as financial, vocational, relationship or medical issues. Some family members felt that the CMr could have made more of an effort to reach out, and would have preferred face-to-face contact over phone. Participants who would not recommend CM to others (6 PwABI and 2 family members at endpoint) said it was unhelpful, time consuming and caused frustration. Some participants would have liked to have a CMr earlier after their injury and were unsure what to expect at the start. Some switched CMrs as their initial CMr stopped and did not have the same connection with the new CMr.

CMrs were very positive about their roles, they specifically appreciated the independence and flexibility in their role to support participants and allocate the time necessary. They found it valuable to stay engaged in cases where they identified potential issues before participants themselves did, and to intervene as soon as participants realized they needed help. Working in teams of two CMrs per case was seen as beneficial, as it provided different perspectives and allowed for dividing support between the person with ABI and their loved ones. Additionally, CMrs valued the opportunity to provide support to family, as this type of support is often unavailable from regular care services. See quote 11.

CMrs sometimes had difficulty determining when to delegate care to another care professional, such as when they believed it was more convenient or efficient to handle it themselves. Some also questioned the sustainability of monitoring clients and accepting new cases in the long term, and if their independence and flexibility would persist beyond the project. See quote 12.

### Reach

All recruited participants were supported by a CMr, as they presented with a need for help at the start of the study. The COVID-19 pandemic created barriers and limited home visits, especially in 2020. Lock-downs and restrictions mostly determined the frequency, intensity and kind of contact which are not related to CM.

### Recruitment

Participants were recruited through professional networks as well as through the sharing of flyers, newsletters and updates on social media. Recruitment was facilitated by CMrs with large professional networks. As professionals saw positive outcomes for their clients through CM, regular referrals followed. Using this procedure, it is not possible to state whether all PwABI with unmet needs where reached.

CMrs were recruited through a formal application process and the project team’s positive relationships with institutions in the region, who were willing to second an employee to CM on a part time basis (usually 4–8 hours per week). Barriers to recruitment of CMrs were encountered in a region where the brain injury network was less well-organized. To keep CMrs engaged, regional and joint meetings were held where they shared experiences, learned new techniques, and discussed the future of CM. Particular emphasis was placed on ongoing reflection on their professional identity and role as CMr. Barriers to engagement for CMrs were difficulty balancing CM with their other job or with navigating the flexibility of their role.

### Context

#### Independence and flexibility

CMrs were employed by an independent foundation created for this project and supervised by project leaders who operate independently from healthcare organizations or institutions. This independence allowed them to act in the best interest of the PwABI and their family without being restricted by organisational protocols, time constraints, or available disciplines within an organisation. Their actions were funded by the project and participants were not charged. The independence and freedom required CMrs to have flexibility and sufficient working experience to determine the optimal approach in various situations.

#### Diverse backgrounds

CMrs valued the diverse professional backgrounds within the team, providing them with easy access to expertise in areas such as job reintegration, psychology, social work, and medical care, as well as lived experiences. However, at times, CMrs struggled with this diversity and wondered if they had missed certain issues in their cases that CMr with a different background could have identified based on their professional experience in another field. See quotes 13 and 14.

The project team, consisting of three members, collaborated on both practical and conceptual aspects of CM. One member was less familiar with care networks, providing a different perspective. The team felt it was effective to have individuals with connections to care organizations in the regions. However, having multiple people from the same organization posed challenges for independence. CMrs appreciated the support and trust from the project team, but sometimes struggled with the freedom in their role. They felt comfortable discussing any concerns with the project team.

#### Regular care

According to the project team and CMrs, good communication about the roles and boundaries of CMs is important for clarifying the CMs’ role as coordinators, not replacements, for other professionals. Placing the client’s needs at the center of discussions with stakeholders helps in this regard. However, the use of different communication systems by various organizations can pose barriers to coordination of care.

The project team notes that the success of CM is influenced by the level of cooperation among regional institutions. When the long-term care system in a region is well-organized for different types of brain injury, professionals seem to better recognize the gaps in psychosocial care and to see the value of case management. On the other hand, when only medical care after stroke is well-organized, it may be challenging to establish support as medical professionals may not fully recognize the need for continuity of psychosocial care for both stroke and other types of ABI. A lack of relationship therapists knowledgeable about ABI was identified as a gap in regular care by CMrs.

## Discussion

In this process evaluation we investigated the feasibility of case management for people with long-term problems on multiple life domains that were not yet well-addressed. Case management proved to be feasible as it was delivered as intended and that the intended participants were reached. The majority of PwABI and their families were satisfied with CM and felt that the support of the CMr was beneficial, and to CMrs the opportunity to provide ongoing support and assist families was of added value.

This process evaluation showed that CM was provided according to CM actions and definitions [16,17]. CMr and participant reports revealed that CMrs supported self-management as much as possible and referred to regular care services rather than providing care themselves. CMrs maintained the professional relationship beyond addressing immediate problems, either through regular check-ins or agreeing that participants could reach out at any time. CMrs viewed their continuous involvement as the surplus value of case management, as it allowed them to follow the PwABI’s and family’ process in recognizing and acknowledging needs. Some participants felt comforted and relieved to have a CMr to rely on and help navigate the healthcare system if new needs arose. This aligns with research advocating for continuous support due to fluctuating needs over time [4], and meets PwABI’s needs for a designated contact person to access care services [18,19].

The average satisfaction rating of just over 7 was the result of mixed evaluations. One third of the PwABI and almost half of the loved ones rated the case manager (CM) with a nine or higher. However, a quarter of the PwABI and their family members gave ratings below five. Those who gave the highest ratings valued the support and personal contact they received. Some indicated that they no longer felt alone in their search for support and that they felt relief as a result. On the other hand, a few participants were disappointed and frustrated. They often attributed their dissatisfaction to a lack of communication and personal involvement from the CM, as well as receiving insufficient support. This could be due to differences in CMr availability, communication style, professional background or lack of a personal connection. Lower satisfaction is not uncommon among people with complex problems and unfavourable social and vocational outcomes. Care providers may be perceived as pessimistic or uncaring for setting realistic goals, and resentment can arise when they do not meet participants’ expectations [20]. The negative experiences indicate that there is room for improvement in delivering CM and highlight the need for clear communication about the role of the CM and what type and amount of support to expect. Important aspect to consider is the continuity of CM over longer periods as new problems and needs may arise at any time, even 20 years later. The independence of care providers was rated positive, as was the diversity of the disciplines in the CMr team combing expertise in different life domains. Since the CMrs worked in teams and met regularly, it is not possible to determine whether the CMr background influenced outcomes.

A content analysis of CM in the Australian National Disability Insurance Scheme (Lukersmith et al., 2021) showed that there was vagueness and ambiguity in the CMr roles and actions leading to poor role communication. As discussed above, this seems to be similar in our study. A clear description of the CMr roles, responsibilities and actions seems essential.

Strengths of this study include its thorough evaluation using both quantitative and qualitative data from participants, CMrs, and the project team. Some limitations need to be mentioned as well. PwABI and their relatives were not involved in the development of CM, nor in the design of the study and interpretation of the results. Experience-based co-design improve the design and therefore the quality of health care services [21]. The relatively low response rate to the evaluation forms and invitations for interviews could lead to a non-response bias if experiences of non-responders differ from those of responders. We did not keep with our exclusion criteria and included people with comorbid neurodegenerative disease or without objectified brain injury after various intakes of people experiencing complaints and problems that were highly likely to be related to ABI (because of an injury/incident in the past even though a formal diagnosis was not given at the time), but for whom no other help was available. In this trial all CMr activities were paid for with project funding, allowing for more freedom in CMr actions and time than is likely to be feasible outside the project.

To the best of our knowledge, there are no prior process evaluations conducted on the feasibility of CM. We adopted the practical framework of Saunders and colleagues [12]. We believe that this was an appropriate choice because the project concerned a pilot to develop CM for PwABI. Implementation science has, however, moved forward and many theories, models and frameworks are now available [22]. More rigorous approaches with stronger theoretical foundations applying appropriate frameworks from implementation science should be followed in case the Dutch Ministry of Health, Welfare, and Sport decides to deploy CM for PwABI as regular care in the Netherlands.

## Conclusion

This process evaluation showed that case management is feasible for people with problems in multiple life domains. Key strengths of CM were the personal and continuous involvement of the CMr, support for family, and the diverse expertise and knowledge within CM teams. Attention should be paid to clear communication about the roles and boundaries of the actions of the CMr. Future research should apply the appropriate implementation science frameworks and involve patients and families to ensure their needs are met.

## Additional File

The additional file for this article can be found as follows:

10.5334/ijic.8650.s1 Supplementary Material. Supplementary Tables 1 to 3 and Figure 1.
