# Supplementary material for: Case Management for People with Acquired Brain Injury with Complex Problems (Part 2): Process Evaluation of a One-Group Trial
Source: Int J Integr Care. 2025 Jul 7;25(3):6. doi: 10.5334/ijic.8650 (PMC12247791; doi:10.5334/ijic.8650)

Supplementary Material

Supplementary Table 1. Type of support offered by the CM, according to participants (n (%))

Question	6 months		Endpoint (18-24 months)	
	**PwABI (n=40)**	**Family members (n=25)**	**PwABI (n=28)**	**Family members (n=13)**
Offered a listening ear (I was able to tell my story)	35 (87,5)	22 (88,0)	21 (75,0)	9 (69,2)
Offered information and advise	31 (77,5)	22 (88,0)	18 (64,3)	9 (69,2)
Helped prepare appointments with external parties (e.g., health care providers, UWV, municipality, health insurers, zorgkantoor, CIZ)	21 (52,5)	12 (48,0)	9 (32,1)	6 (46,2)
Helped me gain insight into my own situation and need for help	20 (50,0)	14 (56,0)	14 (50,0)	5 (38,5)
Provided practical support (e.g., help with handling things)	19 (47,5)	13 (52,0)	11 (39,3)	6 (46,2)
Helped find and/or apply for care or support	17 (42,5)	18 (72,0)	11 (39,3)	7 (53,8)
Went along to appointments with external parties (e.g. care providers, UWV, municipality, health insurer, zorgkantoor, CIZ)	15 (37,5)	10 (40,0)	11 (39,3)	6 (46,2)
Gave information about the brain injury to other caregivers or organizations	13 (32,5)	10 (40,0)	9 (32,1)	2 (15,4)
Provided psychosocial support (e.g., helped me cope with my problems)	11 (27,5)	11 (44,0)	8 (28,6)	3 (23,1)
Helped coordinate various forms of care and assistance	11 (27,5)	13 (52,0)	9 (32,1)	4 (30,8)
Represented my interests	11 (27,5)	9 (36,0)	10 (35,7)	3 (23,1)
Helped me in another way	10 (25,0)	6 (24,0)	6 (21,4)	2 (15,4)
Organized a customized solution	9 (22,5)	7 (28,0)	7 (25,0)	3 (23,1)

Supplementary Table 2. Satisfaction with case management

	6 months		Endpoint (18-24 months)	
	**PwABI (n=40)**	**Family members (n=23)**	**PwABI (n=26)**	**Family members (n=13)**
Rate; mean (standard deviation), range (1-10)	7,50 (1,62), 4-10	7,76 (2,16), 1-10	7,14 (2,63), 1-10	7,46 (2,47), 1-10

Would recommend to others; n (%)	35 (87,5)	21 (91,3)	20 (76,9)	11 (84,6)
Suggestions for improvement; n (%)	13 (32,5)	8 (33,3)	9 (33,3)	3 (23,1)

Supplementary Table 3. Experiences with case management (n (%))*

	**6 months**						**Endpoint (18-24 months)**					
	**PwABI (n=39)**			**Family members (n=24)**			**PwABI (n=27)**			**Family members (n=13)**		
	disagree	neutral	agree	disagree	neutral	agree	disagree	neutral	agree	disagree	neutral	agree
I (have) benefit(ed) from the support provided by the case manager	3 (7,7)	8 (20,5)	28 (71,8)	2 (8,4)	4 (16,7)	18 (75,0)	5 (18,5)	6 (22,2)	16 (59,3)	2 (15,4)	2 (15,4)	9 (69,2)
I felt at ease (with my CM)	1 (2,5)	6 (15,0)	33 (82,5)	1 (4,2)	3 (12,5)	20 (83,3)	4 (14,8)	5 (18,5)	17 (63,0)	2 (15,4)	2 (15,4)	9 (69,2)
I had/have the feeling that I can discuss anything with my case manager	1 (2,6)	10 (25,6)	28 (71,8)	3 (12,5)	3 (12,5)	18 (75,0)	4 (14,8)	5 (18,5)	17 (63,0)	3 (23,1)	2 (15,4)	8 (61,5)
My case manager has enough time for me	3 (7,7)	6 (15,4)	30 (77,0)	4 (16,7)	2 (8,3)	18 (75,0)	3 (11,1)	5 (18,5)	18 (66,7)	2 (15,4)	3 (23,1)	8 (61,5)
My case manager is knowledgeable about brain injury	2 (5,2)	10 (25,6)	17 (69,3)	2 (8,4)	4 (16,7)	18 (75,0)	1 (3,7)	9 (33,3)	16 (59,3)	2 (15,4)	3 (23,1)	8 (61,5)

*Categories ‘strongly disagree’ and ‘disagree’ were collapsed, as were ‘strongly agree’ and ‘agree’.

Supplementary figure 1. Mean number of hours invested by CMrs over 24 months.

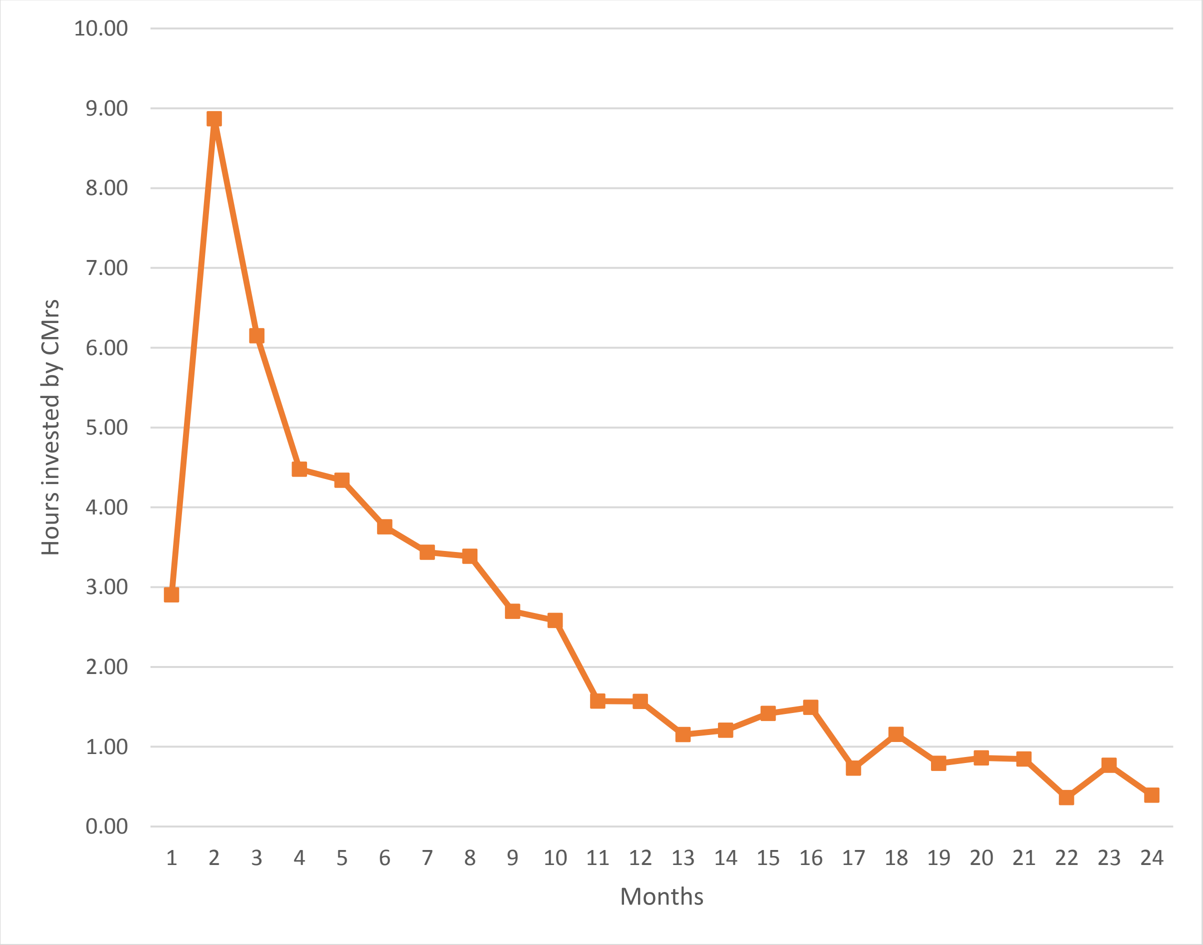

Supplement: Supplementary Material. — Supplementary Tables 1 to 3 and Figure 1. [file ijic-25-3-8650-s1.pdf]
